# Evaluation of Geenius HIV-1/2 Confirmatory Assay for the confirmatory and differential diagnosis of HIV-1/HIV-2 in Japan and reliability of the Geenius Reader in the diagnosis of HIV-2

**DOI:** 10.1186/s12879-021-06291-5

**Published:** 2021-06-14

**Authors:** Shigeru Kusagawa, Ai Kawana-Tachikawa, Keiji Matsubayashi, Yuji Hoshi, Ken Ishimaru, Isao Hamaguchi

**Affiliations:** 1 AIDS Research Center, National Institute of Infectious Diseases, Tokyo, Japan; 2 Central Blood Institute, Blood Service Headquarters, Japanese Red Cross Society, Tokyo, Japan; 3 Blood Service Headquarters, Japanese Red Cross Society, Tokyo, Japan; 4 Department of Safety Research on Blood and Biological Products, National Institute of Infectious Diseases, Tokyo, Japan

**Keywords:** HIV confirmatory test, HIV-1/HIV-2 differentiation test, Sensitivity, Cross-reactivity

## Abstract

**Background:**

NEW LAV BLOT I and II (LAV I and LAV II), they were only option for human immunodeficiency virus (HIV) confirmatory test, following HIV screening test using HIV Ag/Ab combination test in Japan. We evaluated the performance of Geenius HIV-1/2 Confirmatory Assay (Geenius), both as a confirmatory test and for differentiation between HIV-1 and HIV-2, in comparison with LAV I and LAV II.

**Methods:**

Eighty-nine HIV-1-positive plasma specimens, one anti-HIV-1 low-titer performance panel, 10 seroconversion panels, and two anti-HIV-1/2 combo performance panels were tested. The results were read with the Geenius Reader and by visual reading.

**Results:**

All 89 HIV-1-positive plasma specimens were identified as HIV-1-positive using Geenius; this 100% success rate was superior to that with LAV I (95.5% using WHO criteria, 98.9% using CDC criteria). The HIV-1-positive specimens showed low cross-reactivity with HIV-2 lines in Geenius. The sensitivity of Geenius for HIV-1 detection was the same as or greater than that of LAV I, but less than that of Genscreen HIV Ag-Ab ULT, in our analysis of the commercial performance and seroconversion panels. In contrast, five of the 13 HIV-2-positive specimens that had been identified as HIV-positive untypable by visual reading because of their cross-reactivity to HIV-1 lines were successfully identified by the Geenius Reader as HIV-2-positive with cross-reactivity.

**Conclusions:**

Geenius provides strong performance for HIV confirmatory tests and HIV-1 differentiation tests. However, when visual reading is used, its performance in HIV-2 differentiation is less reliable. Because HIV-2 infection has been sporadically reported in Japan, the use of the Geenius Reader is preferable to ensure more reliable HIV-1/HIV-2 differentiation.

## Background

In Japan, specimens that are detected as human immunodeficiency virus (HIV)-positive via the screening test, using HIV Ag/Ab combination test, are subjected to a confirmatory test using western blot test. The confirmatory test kits, NEW LAV BLOT I and II (LAV I and LAV II), have been used for the last 30 years.

As sporadic cases of HIV-2 infection have been reported in Japan since the 2000s [1–3], the roles of LAV I and LAV II have become more important for HIV-1/HIV-2 differentiation. However, it is difficult to diagnose HIV-1 or HIV-2 infection based on the results of the LAV I and LAV II tests alone because of cross-reactivity to anti-HIV-2/anti-HIV-1 antibodies, respectively. In our previous study, 12 of 89 (13.5%) of HIV-1-positive specimens, identified using LAV I and COBAS AmpliPrep/COBAS TaqMan HIV-1 Version 2.0 (Roche Molecular Systems, Inc., NJ, USA), were identified as HIV-2-positive by LAV II testing, and 6 of 13 (46.2%) of HIV-2-positive specimens were identified HIV positive untypable because of the cross-reaction to Env gp160 and Gag p24 of LAV I [4]. According to some reports, HIV-2 infections are often misclassified as HIV-1 while using the HIV-1 western blot [5–7]. In addition, the process of western blotting is complicated, takes a long time, and requires adequate skill in interpreting the results.

The Geenius HIV-1/2 Confirmatory Assay (Geenius) was developed as a confirmatory/supplementary test to improve the diagnostic accuracy of HIV infection. Although the introduction of Geenius in Japan was later than that in American/European countries, it has been available since September 2020. In the US, Geenius has been approved by the FDA and is recommended as a supplementary immunoassay in HIV laboratory testing [8].

In this study, we evaluated Geenius as both a confirmatory test and a differentiation test for HIV-1/HIV-2.

## Methods

### HIV-1-positive specimens

Eighty-nine HIV-1-positive plasma specimens that had been declared ineligible for transfusion were provided by the Japanese Red Cross Blood Centers from 2013 through 2015. The specimens were evaluated using cobas TaqScreen HIV on cobas s 401 (Roche Molecular Systems, Inc., NJ, USA) or Procleix Ultrio Elite ABD assay on Procleix PANTHER System (Grifols Diagnostic Solutions, CA, USA), and decided HIV-1-positive by the discriminatory assay. The specimens were provided following an application for the use of blood donated in Japan, based on the guidelines on the use of donated blood in research and development. The information of the specimens was anonymized, and a decoding index was not created. Ethical approval was obtained from the Ethical Committee of the National Institute of Infectious Diseases (No. 1082).

### Commercial performance panels and seroconversion panels

Anti-HIV-1 Low Titer Performance Panel PRB107 (SeraCare Life Sciences) and 10 seroconversion panels, namely PRB908, PRB911, PRB913, PRB918 (SeraCare Life Sciences), HIV9012, HIV9015, HIV9032, HIV9077, HIV9079, and HIV12008 (Zeptometrix, Buffalo, NY, USA), were used to evaluate the sensitivity of the assays. Anti-HIV-1/2 Combo Performance Panels PRZ201 and PRZ202 (SeraCare Life Sciences, Milford, MA, USA) were used to evaluate the sensitivity, specificity, and cross-reactivity of the assays between anti-HIV-1 and anti-HIV-2.

### Diagnostic HIV tests

The assays thus evaluated were NEW LAV BLOT I, NEW LAV BLOT II (LAV I and LAV II, Bio-Rad Laboratories, Hercules, CA, USA), and Geenius HIV 1/2 Confirmatory Assay (Geenius, Bio-Rad Laboratories). Each assay was performed according to the manufacturer’s instructions. The results of the LAV I assay were primarily interpreted according to the WHO criteria and also according to the CDC criteria [9], as necessary. In brief, two of three Env proteins were required for interpretation of HIV-1-positive in WHO criteria, and two of three proteins, Env gp160/120, Env gp41 and Gag p24, were required for interpretation of HIV-1-positive in CDC criteria. In LAV II test, when one or more Gag, Pol and Env bands were observed, the specimen was interpreted HIV-2-positive. Geenius was read using a Geenius Reader and visual reading according to the instructions in the package insert. In short, when one Env and one or more other HIV-1-specific line(s) were observed, the specimen was decided HIV-1-positive. When two HIV-2-specific lines were observed, the specimen was decided HIV-2-positive. Genscreen ULTRA HIV Ag-Ab (GS ULT, Bio-Rad Laboratories) was used to examine the anti-HIV-1 low titer performance panel and seroconversion panels.

## Results

### Examination of HIV-1-positive specimens

Eighty-nine HIV-1-positive specimens were examined using LAV I and interpreted according to the WHO criteria as recommended in the package insert. This method identified 85 of the specimens (95.5%) as HIV-1-positive (Table 1). When the CDC criteria were applied, the detection of Env gp160 and Gag p24 in three of the four indeterminate specimens caused them to be identified as HIV-1-positive, leading to a total of 88 HIV-1-positive specimens (98.9%) (Table 1). The Geenius assay, by contrast, identified all 89 specimens as HIV-1-positive (Table 1), whether the results were read using Geenius Reader or by visual reading.

**Table 1:** Comparison of LAV I with Geenius Assay for HIV-1-positive specimens in Japan

		LAV I (WHO)			LAV I (CDC)		
		POS*	IND‡	NEG¶	POS*	IND‡	NEG¶
Geenius	HIV-1 POS	85	4	0	88	1	0
	HIV NEG	0	0	0	0	0	0
	Others	0	0	0	0	0	0

POS*: Positive; *IND*‡: Indeterminate; *NEG*¶: Negative

The frequency of each HIV-specific line detected using Geenius is depicted in Table 2. HIV-1 gp160 and gp41 were detected in all tested specimens. The detection frequencies of p31 and p24 were 51.7%. The rates of cross-reactivity to two HIV-2 lines were low (1.1 and 4.5%), and no specimens reacted with both HIV-2 lines at the same time.

**Table 2:** Frequency of HIV-specific line for HIV-1-positive specimens in Japan

	Geenius					
	HIV-2		HIV-1			
	gp36	gp140	p31	gp160	p24	gp41
+	1	4	46	89	46	89
–	88	85	43	0	43	0
+ Rate (%)	1.1	4.5	51.7	100	51.7	100

### Detection sensitivity of LAV I and Geenius

The detection sensitivities of LAV I and Geenius were examined. The results of these assays when tested on the anti-HIV-1 Low Titer Performance Panel PRB107 are shown in Table 3. GS ULT identified 14 of the 15 specimens in the panel as HIV-positive (Table 3). These 14 specimens were decided HIV-1-positive by FDA-approved HIV-1 RNA test results according to the datasheet. Six specimens were identified as HIV-negative by LAV I; the same six were also identified as HIV-negative by Geenius (Table 3 (A), (B)). In LAV I, no HIV-1-positive results were obtained when the WHO criteria were used (Table 3 (B)); however, when the CDC criteria were used, two specimens were identified as HIV-1-positive because of the detection of Env gp160 and Gag p24 (Table 3 (C)). In Geenius, five specimens, including the two mentioned above, were identified as HIV-1-positive (Table 3 (A)) using both the Geenius Reader and visual reading.

**Table 3:** Examination using the anti-HIV-1 Low Titer Performance Panel PRB107

(A) Geenius HIV 1/2 Confirmatory Assay			
		**Genscreen ULTRA HIV Ag-Ab**	
		POS*	**NEG¶**
Geenius	HIV-1 POS*	5	0
	HIV-1 IND‡	3	0
	HIV NEG¶	6	1
	Others	0	0
**(B) NEW LAV BLOT I (WHO criteria)**			
		**Genscreen ULTRA HIV Ag-Ab**	
		POS*	**NEG¶**
LAV I (WHO)	POS*	0	0
	IND‡	8	0
	NEG¶	6	1
**(C) NEW LAV BLOT I (CDC criteria)**			
		**Genscreen ULTRA HIV Ag-Ab**	
		POS*	**NEG¶**
LAV I (CDC)	POS*	2	0
	IND‡	6	0
	NEG¶	6	1

POS*: Positive; *IND*‡: Indeterminate; *NEG*¶: Negative

Table 4 shows the measurement using the seroconversion panels. The bleed numbers indicate the blood samples that were first identified as HIV-1-positive. Geenius was able to return HIV-1-positive results earlier than LAV I with the WHO criteria but later than LAV I with the CDC criteria (Table 4). The detection sensitivity of these kits was less than that of GS ULT (Tables 3 and 4).

**Table 4:** Examination using anti-HIV-1 seroconversion panels

Panel		Member of first HIV-1 detection			
ID	Bleed	GS ULT	Geenius	LAVI (WHO)	LAVI (CDC)
PRB908	01–06	06	06	06	06
PRB911(M)	01–10	02	08	ND	06
PRB913	01–02	02	02	02	02
PRB918	01–06	01	04	04	03
HIV9012	01–08	06	ND^¥^	ND	08
HIV9015	01–10	07	ND	ND	10
HIV9032	01–14	09	14	ND	10
HIV 9077	01–28	12	15	27	15
HIV9079	01–25	09	14	19	13
HIV12008	01–13	09	12	ND	11

ND¥: Not decided “HIV-1 positive”

### Examination of anti-HIV-1/2 combo performance panels

The anti-HIV-1/2 Combo Performance Panels were examined using Geenius. The panel includes 13 HIV-1 positive specimens, 13 HIV-2 positive specimens, 1 indeterminate specimen, and 3 negative specimens. The frequency of each HIV-specific line detected by Geenius is shown in Table 5. HIV-1-positive specimens showed no cross-reactivity to HIV-2-specific lines (Table 5 (A)), whereas approximately 50% of the HIV-2-positive specimens were cross-reactive to four HIV-1-specific lines (Table 5 (B)). The results obtained by visual reading were the same as those using the Geenius Reader.

**Table 5:** Frequency of HIV-specific line in anti-HIV-1/2 Combo Performance panels

(A) HIV-1-positive specimens						
	**Geenius (read by Geenius Reader)**					
	**HIV-2**		**HIV-1**			
	**gp36**	**gp140**	**p31**	**gp160**	**p24**	**gp41**
+	0	0	4	10	3	11
–	13	13	9	3	10	2
+ Rate (%)	0	0	30.8	76.9	23.1	84.6
**(B) HIV-2-positive specimens**						
	**Geenius (read by Geenius Reader)**					
	**HIV-2**		**HIV-1**			
	**gp36**	**gp140**	**p31**	**gp160**	**p24**	**gp41**
+	13	12	6	7	7	7
–	0	1	7	6	6	6
+ Rate (%)	100	92.3	46.2	53.8	53.8	53.8

The Geenius assay successfully identified 10 of the 13 HIV-1-positive specimens as HIV-1-positive and 11 of the 13 HIV-2-positive specimens as HIV-2-positive, including five that were HIV-2-positive with cross-reactivity (Table 6). The five specimens that were HIV-2 positive with cross-reactivity were identified as “HIV-positive untypable” by visual reading because of their cross-reactivity with HIV-1 lines (Table 6). One HIV-2-positive specimen was misidentified as HIV-1-positive, since HIV-2 gp140 was not detected (Table 6).

**Table 6:** Examination of anti-HIV-1/2 Combo Performance panels

Geenius (read by Geenius Reader)	Datasheet			
	HIV-1 POS*	HIV-2 POS	HIV IND‡	HIV NEG¶
HIV POS U†	0	1	0	0
HIV-1 POS	10	1	0	0
HIV-2 POS	0	6	0	0
HIV-2 POS W$	0	5	0	0
HIV IND	0	0	0	0
HIV-1 IND	1	0	0	0
HIV-2 IND	0	0	0	0
NEG	2	0	1	3

*HIV POS U*†: HIV-positive untypable; *HIV-2 POS W*$: HIV-2 positive with cross-reactivity, classified “HIV POS U” by visual reading; POS*: Positive; *IND*‡: Indeterminate; *NEG*¶: Negative

## Discussion

Our study demonstrated the usefulness and characteristics of Geenius. Eighty-nine HIV-1-positive specimens collected in Japan were identified as HIV-1-positive using the Geenius assay, demonstrating a successful detection rate superior to that with LAV I. The detection frequencies of Env gp160, gp120, gp41 and Gag p24 bands were 88/89 (98.9%), 85/89 (95.5%), 81/89 (91.0%), and 89/89 (100%), respectively, with LAV I in our previous study [4]. In contrast, the detection rate of two HIV-1 Env bands, which are important for HIV-1 diagnosis, was 100% using Geenius. In addition, 12 of these specimens were identified as HIV-2-positive by LAV II testing because of the cross-reactivity (12/89, 13.5%) [4]. Cross-reactivity to Gag p26 (85/89, 96.6%), Pol p34 (38/89, 42.7%), Pol p68 (27/89, 30.3%), and Env gp105 (21/89, 23.6%) were frequently observed [4]. Geenius showed low cross-reactivity with HIV-2 lines in the examination of the HIV-1-positive specimens.

Geenius exhibited an HIV-1 detection sensitivity comparable to or greater than that of LAV I but lower than that of GS ULT when the results of LAV I were interpreted according to the WHO criteria. Montesinos et al. reported that five of 11 specimens from the acute phase of HIV-1 infection were identified as HIV-1-positive using Geenius [10]. Abbate et al. [11] and Wong et al. [12] reported that the sensitivity of the test for acute HIV-1 infection was low. Kondo et al. reported that seven of 20 specimens from the acute phase of HIV-1 infection were identified as HIV-1-positive [13]. In addition, two of 130 HIV-negative specimens and two of 10 Determine HIV-1/2 (Alere Medical, Chiba, Japan)-positive and HIV-1 nucleotide amplification test-negative (HIV pseudo-positive) specimens cross-reacted with HIV-1-specific lines [13]. These findings indicate that Geenius should be used for confirmatory/supplementary testing of specimens that tested positive in HIV screening tests.

It has been reported that 46 to 85% of HIV-2-positive cases in the US were misdiagnosed as HIV-1-positive, based on HIV-1 western blot analysis [5–7]. When HIV-2-positive specimens included in the anti-HIV-1/2 Combo Performance Panels, PRZ201 and 202, were tested using LAV I, all 13 specimens were determined HIV-1-indeterminate using LAV I with the WHO criteria. However, the results of six of these specimens (46.2%) were changed to HIV-1-positive upon interpretation with the CDC criteria because of their cross-reactivity to Env gp160 and Gag p24 [4]. Although the detection sensitivity of LAV I with the CDC criteria was higher than that with the WHO criteria, the former was not recommended for discrimination between HIV-1 and HIV-2 infection.

The cross-reactivity of anti-HIV-2 antibodies to HIV-1 antigens was also observed in the Geenius assay. Six of the 13 HIV-2-positive specimens were identified as HIV-positive untypable by visual reading was used because of cross-reactivity with HIV-1 lines. Five of these six were identified as HIV-2-positive with cross-reactivity when the Geenius Reader was used because the reader could discriminate between specific reactions and cross-reactions of anti-HIV-2 antibodies with HIV-1 lines. The positive agreement rate of HIV-2-positive specimens was 6/13 (46.2%) by visual reading and 11/13 (84.6%) by the Geenius Reader. Herssens et al. reported that six of 10 HIV-2-positive specimens were identified as HIV-positive untypable when visual reading was used in the Geenius assay [14]. In contrast, when the Geenius Reader was used, Malloch et al. reported that 52 out of 53 (98.1%) HIV-2-positive specimens were identified as HIV-2-positive [15], while Montesinos et al. reported that four out of five HIV-2-positive specimens were identified as HIV-2-positive [10]. These results indicate that Geenius offers strong performance for HIV-2 diagnosis when it is used with the Geenius Reader.

One HIV-2-positive specimen (PRZ202–02) was misidentified as HIV-1-positive using Geenius because of the failure to detect HIV-2 Env gp140, although LAV I/II with the WHO criteria was able to identify this specimen as HIV-2 positive [4]. Furthermore, clinical reports from HIV-2 epidemic areas should be regularly observed because our data were limited.

Geenius has an strong performance as an HIV confirmatory test; however, its performance when interpreted with visual reading is a cause for some concern. It is important to differentiate between HIV-1 and HIV-2 infection because HIV-2 has innate drug resistant mutations for non-nucleoside reverse transcriptase inhibitors and some protease inhibitors [16], and the viral load cannot be quantified to monitor the benefit of antiretroviral therapy using universally distributed HIV-1 RNA quantitation kits. Although HIV-2 infection has been reported only sporadically in Japan, the use of the Geenius Reader is preferable to ensure more reliable HIV-1/2 differentiation. The reader can manage a sample with a bar code on a cassette, save digital capture, and interpret the result automatically. It is also useful to minimize the misidentification of samples, to secure traceability, and to minimize variation in interpretation between individuals.

## Conclusions

Geenius has a strong performance for HIV confirmatory tests and HIV-1 differentiation tests; when visual reading is used, however, its performance in HIV-2 differentiation is less reliable. Almost all cases of HIV infection in Japan are caused by HIV-1. To reduce the cost of equipment introduction, some diagnostic laboratories might opt for the visual reading method. However, HIV-2 infection has been sporadically reported in Japan, and correct diagnosis leads to an appropriate choice of treatment. The use of the Geenius Reader is preferable to ensure more reliable HIV-1/HIV-2 differentiation.

## Data Availability

The dataset used in this manuscript is available from the corresponding author upon reasonable request.

## Abbreviations

HIV: Human immunodeficiency virus
LAV: NEW LAV BLOT
WHO: World Health Organization
CDC: Centers for Disease Control and Prevention
FDA: Food and Drug Administration
GS ULT: Genscreen ULTRA HIV Ag-Ab
